# Gynecologic bleeding revealing vaginal metastasis of renal cell carcinoma

**DOI:** 10.11604/pamj.2013.14.62.838

**Published:** 2013-02-14

**Authors:** Zineb Benbrahim, Ali Chouaib, Renaud Mazeron, Marie Bénédicte Leger-Ravet, Catherine Lefort, Catherine Lhommé, Omar El Mesbahi, Bernard Escudier

**Affiliations:** 1 Department of medecine, Gustave Roussy Institute, France; 2 Department of Medical Oncology, Hassan II University Hospital, Morocco; 3 Department of urology, Tenon Hospital, France; 4 Department of Pathology, Long Jumeau Hospital, France

**Keywords:** Gynecologic bleeding, vagina, metastasis, carcinoma

## Abstract

Vaginal metastases of renal cell carcinoma have been rarely described. We report a case of a 75-year old woman, who underwent radical right nephrectomy for a renal cell carcinoma. Tumour was classified pT3bN0M0 and grade III of Furhmann grading. One year later, scanner discovered mediastinal and lombo-aortic lymph nodes. She received 2 months of immunotherapy associated with bevacizumab, but stopped because of intolerance. She was readmitted in our institute for vaginal bleeding. Clinical investigations showed a vaginal mass and biopsy revealed a renal cell carcinoma metastasis. This case suggests that retrograde venous dissemination may be at the origin of vaginal metastasis of renal cell carcinoma and emphasized the preventive value of early ligature of renal vein.

## Introduction

The vaginal metastases of the renal cancer are rare. Eighty five cases were reported in the literature. These secondary locations deserve a particular attention because their diagnosis often precedes that of the Renal Cell Carcinoma. We report the case of a woman who presented vaginal metastasis two years after nephrectomy for a Renal Cell carcinoma and we discuss the modes of dissemination of these metastases.

## Patient and observation

**Figure 1:**
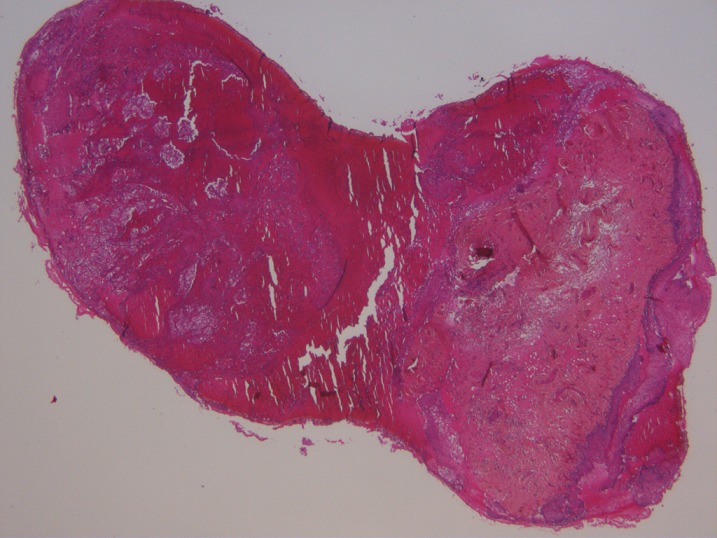
Neoplastic cells in deep chorion (HE x25)

**Figure 2:**
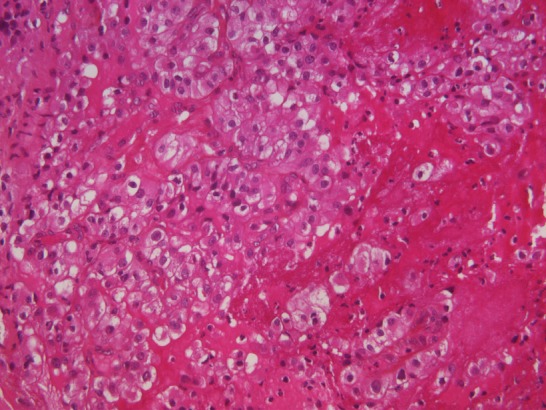
Neoplastic clear cells (HE x 250)

**Figure 3:**
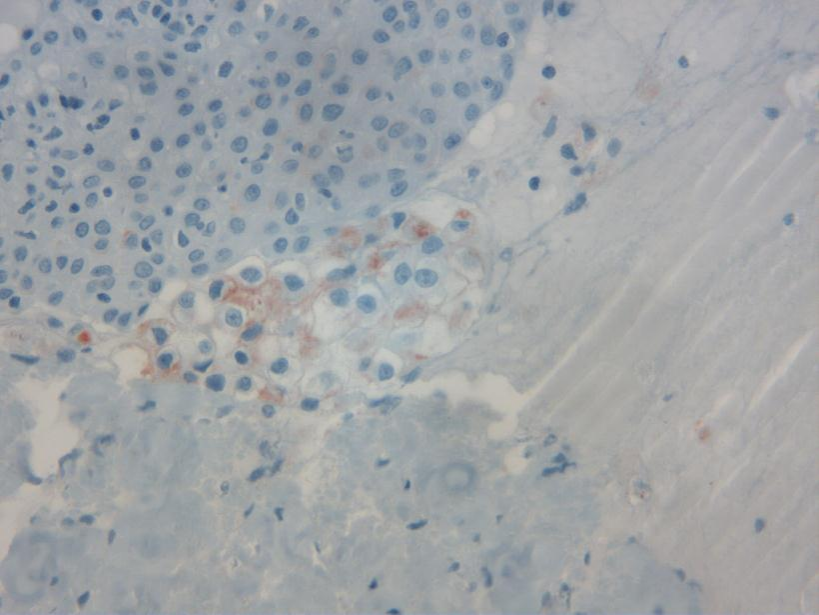
Immunoreactivity of neoplastic cells for CD10 (x250)

A 75-year-old white woman, had history of fullness in the right upper quadrant, and a weight loss. On physical examination there was a large palpable mass in the right upper quadrant. Radiologic examination revealed tumor of right kidney measuring 14 x 8cm. A right nephrectomy was done through a long midline incision. The excised organ measured 15 x 9 x 6.5cm and weighed 660 g. The microscopic appearance was typical of renal cell carcinoma. The tumor was classified grade III of Furhman grading system and pT3b N0 M0 of the Union of International Cancer Control classification. Evolution has been marked by the occurrence of mediastinal and lombo-aortic lymph nodes. She received two months of targeted therapy (bevacizumab associated with interferon) that was stopped because of intolerance. At that time, disease was stable on CT scan and remained stable for almost one year. She was readmitted because of vaginal bleeding. On examination a 4 x 5 cm mass was found arising from the lower one-third of the vagina. A biopsy revealed uniform population of neoplastic cells with clear cytoplasm, atypical nuclei (Figure 1 and Figure 2) and positive staining with KL1 and CD10 (Figure 3). Anatomopathologists concluded to a metastasis of renal cell carcinoma. External hemostatic radiotherapy was started delivering a tumor dose of 20 Gy in 6 cycles. Patient is still alive 6 months later with local control.

## Discussion

Primary adenocarcinoma of the vagina comprises 9% of all vaginal neoplasms [1]. Therefore, adenocarcinoma of vagina must be considered as metastatic until the opposite is demonstrated [2]. This metastasis may develop from the cervix, endometrium, ovary, or colon in approximately 65% of cases [3]. Secondary localisations from the pancreas, stomach, and kidney are even less common [4]. The first description of vaginal metastasis from renal cell carcinoma was reported in 1906 [5]. In 2003, Mendese had reviewed 85 cases of theses tumors. Median age at diagnosis was 57 years (average 14-88). Clinical presentation in 65% of cases was vaginal leaking, haemorrhage, or mass effect. The vaginal lesion size ranged from 0.5 to 8 cm. Renal cell carcinoma vaginal metastasis usually preceded and rarely came after the diagnosis of renal tumor. In 63% of cases, primary location of tumor was left kidney. Usuallly metastasis is solitary. It appears at the same side of primary tumor and is usually located in the lower third of the anterior wall of the vagina [6]. The most important prognostic factor in patients with vaginal metastasis is the presence or absence of other secondary localisations. Moreover, patients with metachronous metastasis present longer survival than those with synchronous metastasis. Median survival of all types confounded is 19 months (range 1- 96) [4, 6, 7].

Several pathways (urinary, lymphatic and systemic) were evoked at the origin of vaginal implantation of renal carcinoma. However, the only one having been demonstrated is the venous retrograde way. Movedlcahy and Furlow objectified radiologically flux between the renal vein and the genital vein at several patients with renal cancer and vaginal metastasis [8].

## Conclusion

This observation suggests the interest of early ligature of genital vein during nephrectomy to limit migration of neoplasmic cell and prevent gynaecologic metastasis.
